# Image-based features in machine learning to identify delivery errors and predict error magnitude for patient-specific IMRT quality assurance

**DOI:** 10.1007/s00066-023-02076-8

**Published:** 2023-03-29

**Authors:** Ying Huang, Yifei Pi, Kui Ma, Xiaojuan Miao, Sichao Fu, Hua Chen, Hao Wang, Hengle Gu, Yan Shao, Yanhua Duan, Aihui Feng, Weihai Zhuo, Zhiyong Xu

**Affiliations:** 1 Shanghai Chest Hospital, School of Medicine, Shanghai Jiao Tong University, 200030 Shanghai, China; 2 Department of Radiation Oncology, The First Affiliated Hospital of Zhengzhou University, Henan, China; 3 Varian Medical Systems No.8 Yun Cheng Street, Beijing, China; 4 The General Hospital of Western Theater Command PLA, Chengdu, China; 5 Key Lab of Nucl. Phys. & Ion-Beam Appl. (MOE), Fudan University, Shanghai, China

**Keywords:** Thoracic cancer, Error prediction, Dose distribuction, Artificial intelligence, Random forest

## Abstract

**Objective:**

To identify delivery error type and predict associated error magnitude by image-based features using machine learning (ML).

**Methods:**

In this study, a total of 40 thoracic plans (including 208 beams) were selected, and four error types with different magnitudes were introduced into the original plans, including 1) collimator misalignment (COLL), 2) monitor unit (MU) variation, 3) systematic multileaf collimator misalignment (MLCS), and 4) random MLC misalignment (MLCR). These dose distributions of portal dose predictions for the original plans were defined as the reference dose distributions (RDD), while those for the error-introduced plans were defined as the error-introduced dose distributions (EDD). Both distributions were calculated for all beams with portal dose image prediction (PDIP). Besides, 14 image-based features were extracted from RDD and EDD of portal dose predictions to obtain the feature vectors. In addition, a random forest was adopted for the multiclass classification task, and regression prediction for error magnitude.

**Results:**

The top five features extracted with the highest weight included 1) the relative displacement in the x direction, 2) the ratio of the absolute minimum residual error to the maximal RDD value, 3) the product of the maximum and minimum residuals, 4) the ratio of the absolute maximum residual error to the maximal RDD value, and 5) the ratio of the absolute mean residual value to the maximal RDD value. The relative displacement in the x direction had the highest weight. The overall accuracy of the five-class classification model was 99.85% for the validation set and 99.30% for the testing set. This model could be applied to the classification of the error-free plan, COLL, MU, MLCS, and MLCR with an accuracy of 100%, 98.4%, 99.9%, 98.0%, and 98.3%, respectively. MLCR had the worst performance in error magnitude prediction (70.1–96.6%), while others had better performance in error magnitude prediction (higher than 93%). In the error magnitude prediction, the mean absolute error (MAE) between predicted error magnitude and actual error ranged from 0.03 to 0.33, with the root mean squared error (RMSE) varying from 0.17 to 0.56 for the validation set. The MAE and RMSE ranged from 0.03 to 0.50 and 0.44 to 0.59 for the test set, respectively.

**Conclusion:**

It could be demonstrated in this study that the image-based features extracted from RDD and EDD can be employed to identify different types of delivery errors and accurately predict error magnitude with the assistance of ML techniques. They can be used to associate traditional gamma analysis with clinically based analysis for error classification and magnitude prediction in patient-specific IMRT quality assurance.

**Supplementary Information:**

The online version of this article (10.1007/s00066-023-02076-8) contains supplementary material, which is available to authorized users.

## Introduction

With the extensive application of intensity-modulated radiation therapy (IMRT) in radiotherapy, the treatment modality provides highly conformal and complex dose distributions through multiple beams. This contributes to forming steep dose gradients to shield critical organs at risk (OARs) from adjacent high-dose regions [1, 2], thus leading to complexity in planning and delivery [3, 4]. Delivery parameters such as gantry, monitor unit (MU), multileaf collimator (MLC) position, and collimator are different from the planned parameters, which will cause dose differences among patients [5]. Therefore, it is required to ensure the accuracy and safety of patient-specific quality assurance (QA) prior to treatment [6–9].

The patient-specific QA is commonly performed by measurement, and, subsequently, dose distribution is evaluated through gamma analysis that combines dose difference (DD) and distance-to-agreement (DTA) [10, 11]. Despite its universal prevalence in the clinical environment, it has been reported that gamma analysis is insensitive to some errors [12–14]. Besides, gamma analysis results show the rate of the number of points that meet established criteria but cannot exhibit the correlation with the clinical dose differences [15]. For failed plans, it is difficult for users to obtain clear insight into the error source, error magnitude, and so forth.

Recently, much attention has been paid to the application of radiomics and ML to the detection and classification of errors in IMRT QA [2, 16–20]. Landon S adopted gamma radiomics to improve the detection of errors [16]. Nyflota et al. used convolutional neural networks to identify MLC errors in radiotherapy delivery by radiomics analysis of gamma images [17]. Madoka et al. detected MLC modeling errors using radiomics-based ML and fluence difference maps [18]. Kimura et al. evaluated the application of dose difference maps with a convolutional neural network (CNN) in an attempt to detect MLC positional errors in patient-specific QA for volumetric modulated radiation therapy (VMAT) [19]. Potter proposed a dual neural network method to achieve simultaneous error detection and classification by extracting the dose difference histogram (DDH) for the low-dose gradient region and two signed DTA maps [20]. Ma et al. performed radiomics analysis on structural similarity (SSIM) sub-index maps and developed ML models to classify delivery errors in patient-specific dynamic IMRT QA [2].

Through the abovementioned studies, it can be demonstrated that radiomics-based methods and ML could be employed to detect errors effectively, which provides a direction for ML and deep learning (DL) in error classification. However, an error classification model with higher accuracy and clinical significance is expected to be established by integrating the following factors: 1) Since the influence of absolute dose and distance cannot be properly balanced by the input based on dose difference, DTA maps, or gamma images, in which partial information between dose and distance may be ignored, the input with more information about dose and distance may be taken into account. 2) Most existing studies are conducted based on error types, without considering the influence of error magnitude. Nithiyanantham et al. analyzed the clinical consequence of MLC positional errors [21]. The results show that the average change of dose D95% to the planning target volume (PTV) for ±1 mm, ±0.5 mm, and ±0.3 mm was 5.15%, 2.58%, and 0.96% for brain cases; 7.19%, 3.67%, and 1.56% for head and neck cases; and 8.39%, 4.5%, and 1.86% for prostate cases, respectively. It was concluded that the average changes in dose increased with the amount of MLC positional error. As dose distribution is related to different levels of error magnitude, it is necessary to perform an investigation of the prediction models containing error types and error magnitude. 3) It is expected that the accuracy of classification will be improved through more effective prediction models.

Our research team has adopted the DL technique to predict dose distribution based on reference dose distributions of portal dose predictions [22]. Furthermore, the above three factors were integrated into this study to predict error types and error magnitude. In addition, different magnitude levels were introduced into the reference plans to simulate the delivery errors. Portal dose (PD) images were calculated for all the beams with and without introduced errors. The dose distribution of original plans was defined as RDD and that of error-introduced plans was defined as EDD. Additionally, the feature vectors that can describe different errors were extracted from the RDD and EDD of portal dose predictions. In most radiomic studies to date, traditional radiomics features have been employed to establish the model, such as the previously described texture features [2, 16, 18]. Different from those studies, the image-based features representing the underlying error types were extracted in this study.

Moreover, the random forest algorithm was adopted to establish a model for error detection and classification in delivery and predict error magnitude under different error types before treatment. This study on error classification and error magnitude prediction based on RDD and EDD of portal dose predictions is expected to improve the accuracy of error detection and provide support for the analysis of the reason for clinically failed plans in the future.

## Materials and methods

### Clinical plans

A total of 40 thoracic IMRT plans (including 208 beams) delivered by a Varian EDGE (TrueBeam; Varian Medical Systems, Palo Alto, CA, USA) linear accelerator equipped with a Millennium 120 MLC from December 2019 to May 2020 were randomly selected. The characteristics of these plans are listed in Table 1. The modulation complexity score (MCS) was selected to quantify plan complexity, which was found to be the most sensitive to delivery and plan parameters [23]. The MCS score ranges from 1 for a simple unmodulated field and decreases towards 0 with increasing inherent plan complexity. The delivery mode for these IMRT plans was step-and-shoot. Patient-specific dose verification was performed prior to treatment using portal dosimetry. Whereas 30 plans were used for model training and validation, the remaining 10 plans were used for testing. All plans were generated in Eclipse (version 11, Varian Medical Systems). The dose distribution of each plan was calculated through Acuros External Beam (AXB, version 11.0.31, Varian Medical Systems), with a dose calculation grid of 2.5 mm.

**Table 1:** Plan characteristics

Plan type	Lung	Postoperative esophageal	Simultaneous integrated boost
No. of patients	25	3	12
No. of fractions	25/30	28	28
Dose (Gy)	50/60	50.4	60.2 and 50.4
No. of fields (median)	5	4	6
Total MU (mean ± SD)	511.48 ± 131.20	378.67 ± 55.41	593 ± 141.95
Volume of PTV (cc)	277.53 ± 139.33	244.88 ± 104.67	188.17 ± 123.21
MCS (mean ± SD)	0.5495 ± 0.08	0.6192 ± 0.10	0.5511 ± 0.08

*MU* monitor unit, *SD* standard deviation, *PTV* planning target volume, *MCS* modulation complexity score

### Error simulation

In the plan delivery, most dose differences originate from collimator angle misalignment, MU variation, systematic MLC misalignment, and random MLC misalignment. In this study, an in-house program based on Python was developed and four types of errors with different magnitude levels were introduced into the original plan. The original parameters in all control points described in the DICOM RT plan file were modified with a specified shift, and the files were imported back into the TPS. The dose distributions of portal images for error-free plans and error-introduced plans were calculated with portal dose image prediction (PDIP; v. 13.5.35, Varian Medical Systems). Gamma analysis between the RDD and EDD of portal dose predictions were performed based on the criteria 3%/3 mm, 3%/2 mm, 2%/3 mm, and 2%/2 mm, with a 10% threshold.

As for the model training and validation, a total of 151 RDD maps and 2567 EDD maps were generated for 30 plans.

#### Error-free plans

Error-free plans comprised the unmodified beam.

#### Collimator error plans

In collimator error plans (denoted COLL error), each control point of the clinical plan was modified to introduce collimator angle errors of 1°, 2°, and 3°. Each clinical plan generated three plans with different collimator error magnitude levels.

#### Monitor unit error

The MU of each control point of the clinical plan was modified by ±1%, ±3%, and ±5%, respectively. Each clinical plan generated six plans with different MU error magnitude levels.

#### Systematic misalignment of MLC

For the systematic misalignment of MLC (denoted MLCS error), all open leaves in both banks were offset by 1 mm, 2 mm, 3 mm, and 5 mm in the same direction at every control point. Each clinical plan generated four plans with different MLCS error magnitude levels.

#### Random misalignment of MLC

For the random misalignment of MLC (denoted MLCR error), all open leaves in both banks were modified by an in-house Python script describing that the distribution of the values follows a Gaussian distribution with a standard deviation of 1 mm, 2 mm, 3 mm, and 5 mm. Meanwhile, the maximum position of the MLC that can be shifted satisfied the constraints of the MLC bracket and the leaf gap to ensure a deliverable position of the leaf. Each clinical plan generated four plans with different MLCR error magnitude levels.

Moreover, the error plans including COLL (1.5°, 2.5°), MU (1.5%, 2.5%), MLCS (1.5 mm, 2.5 mm), and MLCR (1.5 mm, 2.5 mm) were generated for the testing set, and the dose distribution of portal dose predictions for these plans were calculated.

### Feature extraction

By analyzing the generating mechanism of different types of errors, 14 features were extracted from RDD and EDD of portal dose predictions with specific errors, as shown in Table 2.

**Table 2:** Feature description and index

Feature	Definitions	Index
$r_{E/R}$	The ratio between the proportion of the maximum gradient direction to all gradient directions in $G\mathrm{'}$ corresponding to EDD and RDD	(9)
$\hat{\theta }$	The displacement corresponding to the maximum overlap	(10)
$\rho _{\mathit{\max }1}$	The maximum NCC value corresponding to$\hat{\theta }$	(11)
$\hat{d}_{x}$	The relative displacement in the x direction	(12)
$\hat{d}_{y}$	The relative displacement in the y direction	(12)
$\rho _{\mathit{\max }2}$	The maximum NCC value between *T*_*E*_ and *T*_*R*_	(13)
$d_{\mathit{\max }1}$	The maximum residual	(S-2)
$d_{\mathit{\min }1}$	The minimum residual	(S-3)
$d_{\mathit{\max }\times \mathit{\min }}$	The product of the maximum and minimum residuals	(S-4)
$d_{\mathit{mean}1}$	The mean value of residual	(S-5)
$d_{\mathit{\max }2}$	The ratio of the absolute maximum residual error to the maximal RDD value	(S-6)
$d_{\mathit{\min }2}$	The ratio of the absolute minimum residual error to the maximal RDD value	(S-7)
$d_{\mathit{mean}2}$	The ratio of the absolute mean residual error to the maximal RDD value	(S-8)
$d_{\text{ratio}}$	The ratio of the mean residual error to the mean RDD	(S-9)

*NCC* normalized cross correlation

The details of feature extraction are outlined in the following sections.

#### In terms of COLL

In terms of COLL, the relative rotational parameters between EDD and RDD of portal dose predictions were estimated, and the corresponding feature extraction methods that could characterize the rotation were constructed. The steps are presented as follows:

The gradient between EDD and RDD in the y direction can be calculated as follows:

1 $$I_{x}(x{,}y)=I(x+1{,}y)-I(x-1{,}y)$$

2 $$I_{y}(x{,}y)=I(x{,}y+1)-I(x{,}y-1)$$

3 $$G(x{,}y)=\mathit{\arctan }(I_{y}(x{,}y)/I_{x}(x{,}y))$$

where *I*(*x,y*) represents the image matrix of EDD or RDD, respectively, and the subscript x or y denotes the gradient in the respective direction; G represents the gradient direction image, arctan is the four-quadrant inverse tangent function ([$-\uppi {,}+\uppi$]).

To avoid the adverse effects of low-value noise on the statistical results, the gradient threshold is set to 10% of the maximum value of the original image, namely $\mathrm{th}1=0.1\times \max (\mathrm{I})$. These points with corresponding gradients higher than *th1* are selected to form $G\mathrm{'}$:

4 $$G'=\left\{G\left(x{,}y\right)| \left(\begin{array}{ccc} I_{x}(x{,}y)> =\mathrm{th}1 & \mathrm{or} & I_{y}(x{,}y)> =\mathrm{th}1 \end{array}\right)\right\}$$

**Fig. 1:**
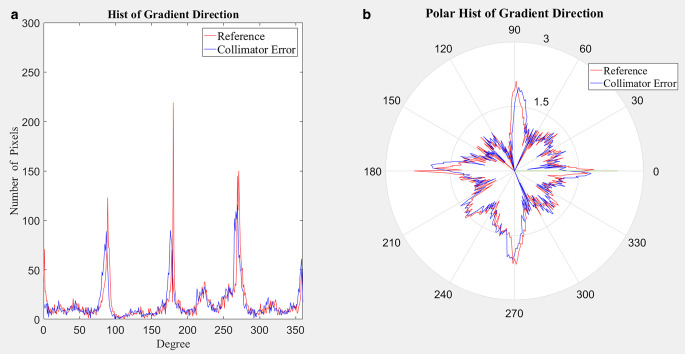
Histograms of the gradient direction. **a** Cartesian coordinate system, **b** polar coordinate system (*red* and *blue* curves represent *H*_*R*_ and *H*_*E*_, respectively. In order to make the curve in the polar coordinate system more intuitive, the polar radius is processed by the common logarithm log10)

The histograms of $\mathrm{G}'$ for EDD and RDD can be calculated as follows,

5 $$H_{R}=\text{hist}({G}_{R}^{'})$$

6 $$H_{E}=\text{hist}({G}_{E}^{'})$$

where ${G}_{R}^{'}$ and ${G}_{E}^{'}$ represent the corresponding ${G}_{*}^{'}$ of RDD and EDD, respectively; “hist” (*) represents histogram statistics; and *H*_*R*_ and *H*_*E*_ represent the histograms of gradient directions corresponding to RDD and EDD, respectively. As for the histogram, 1° is taken as the bin size and there are 360 bins in total. The gradient direction histograms corresponding to RDD and EDD are shown in Fig. 1.

The proportion of the maximum gradient direction to all gradient directions in $G\mathrm{'}$ corresponding to EDD and RDD, and the ratio between these two proportions can be calculated as follows:

7 $$r_{R}=\mathit{\max }(H_{R})/\mathrm{sum}(H_{R})$$

8 $$r_{E}=\mathit{\max }(H_{E})/\mathrm{sum}(H_{E})$$

9 $$r_{E/R}=r_{E}/r_{R}$$

A recurrent shift was performed on *H*_*E*_ within a certain angle range (e.g., ± 10°) to obtain the histogram of *H*_*E*_(*θ*), and the normalized cross correlation (NCC) was calculated with *H*_*R*_. Besides, the displacement of the maximum NCC was searched, which can be used as the estimated value of the rotation angle:

10 $$\hat{\theta }=\underset{\theta }{\textit{argmax}}\left(\mathrm{NCC}\left(H_{E}\left(\theta \right){,}H_{R}\right)\right)$$

11 $$\rho _{\mathit{\max }1}=\mathrm{NCC}\left(H_{E}\left(\hat{\theta }\right){,}H_{R}\right)$$

where $\hat{\theta }$ represents the displacement corresponding to the maximum overlap of the blue curve and the red curve in Fig. 1a, or the rotation angle corresponding to the maximum overlap of the blue curve and the red curve in Fig. 1b. Meanwhile, the maximum NCC value corresponding to $\hat{\theta }$ can also be obtained.

#### In terms of MU error

**Fig. 2:**
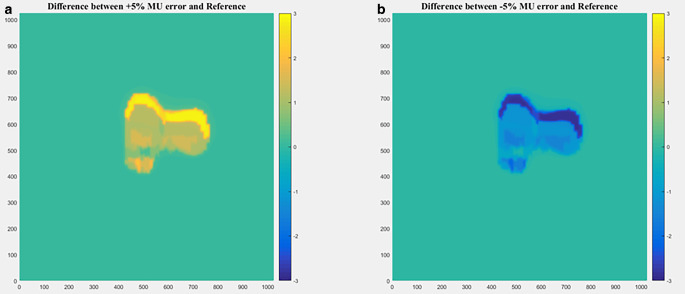
Residual image of monitor unit (*MU*) error. **a** Dose difference with +5% MU error, **b** dose difference with −5% MU error

In terms of MU error, the residual image was obtained by subtracting EDD from RDD, as shown in Fig. 2. The pixel value information of the residual image was counted as the feature to evaluate MU error. The features of $d_{\mathit{\max }1}$, $d_{\mathit{\min }1}$, $d_{\mathit{\max }\times \mathit{\min }}$, $d_{\mathit{mean}1}$, $d_{\mathit{\max }2}$, $d_{\mathit{\min }2}$, $d_{\mathit{mean}2}$, and *d*_*ratio*_ were calculated as shown in the supplementary material.

#### In terms of MLCS error

**Fig. 3:**
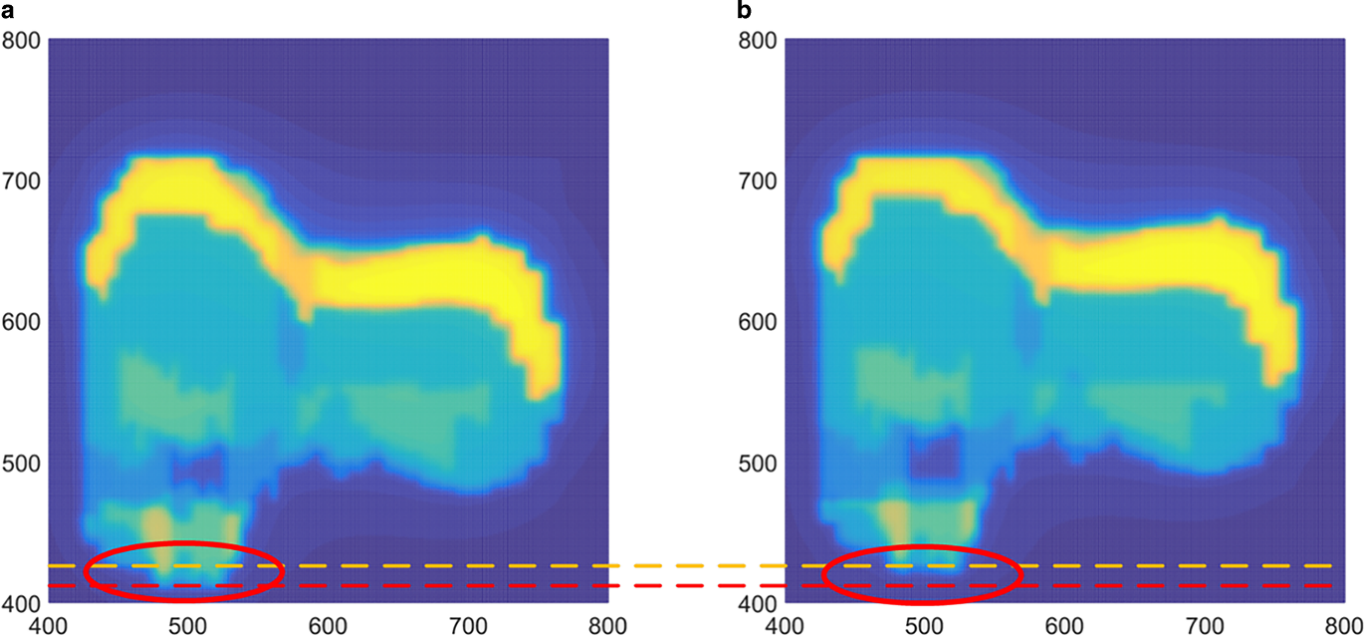
Comparison of the dose distribution of portal dose predictions after partial magnification. **a** Dose distribution with error free, **b** dose distribution with 5 mm multileaf collimator misalignment error

In terms of MLCS error, the EDD is equivalent to the translation of the RDD, as shown in Fig. 3. Therefore, the translation rotation features were extracted to estimate the relative displacement between EDD and RDD. The steps are presented as follows:

The pixel value higher than *th1* in RDD was obtained as the external rectangle. The external rectangle was expanded by two pixels outward in the upper, lower, left, and right directions, and the image template *T*_*R*_ in RDD was cut out. The same size *T*_*E*_ was obtained in EDD. Subsequently, the displacement generating the maximum NCC between *T*_*E*_ and *T*_*R*_ was traversed and searched as the relative displacement estimation between EDD and RDD:

12 $$\hat{d}_{x}{,}\hat{d}_{y}=\underset{d_{x}{,}d_{y}}{\textit{argmax}}\left(\mathrm{NCC}\left(T_{R}{,}T_{E}\left[d_{x}{,}d_{y}\right]\right)\right)$$

13 $$\rho _{\mathit{\max }2}=\mathrm{NCC}\left(T_{R}{,}T_{E}\left[\hat{d}_{x}{,}\hat{d}_{y}\right]\right)$$

where *d*_*x*_ and *d*_*y*_ represent the relative displacement between *T*_*E*_ and *T*_*R*_, and the corresponding maximum NCC value can be obtained.

#### In terms of MLCR error

In terms of MLCR error, no feature extraction method could be employed to accurately characterize the type and magnitude of this error. The type and magnitude of the MLCR error were estimated by combining the feature vectors corresponding to other types of errors.

A series of features extracted as described above was employed to form 14-dimensional feature vectors as follows: $\rho _{\mathit{\max }1}$, *r*_*E*∕*R*_, $\hat{\theta}$, $\rho _{\mathit{\max }2}$, $\hat{d}_{x}$, $\hat{d}_{y}$, $d_{\mathit{\max }1}$, $d_{\mathit{\min }1}$, $d_{\mathit{\max }\times \mathit{\min }}$, $d_{\mathit{mean}1}$, $d_{\mathit{\max }2}$, $d_{\mathit{\min }2}$, $d_{\mathit{mean}2}$, and *d*_*ratio*_.

### Model construction

**Fig. 4:**
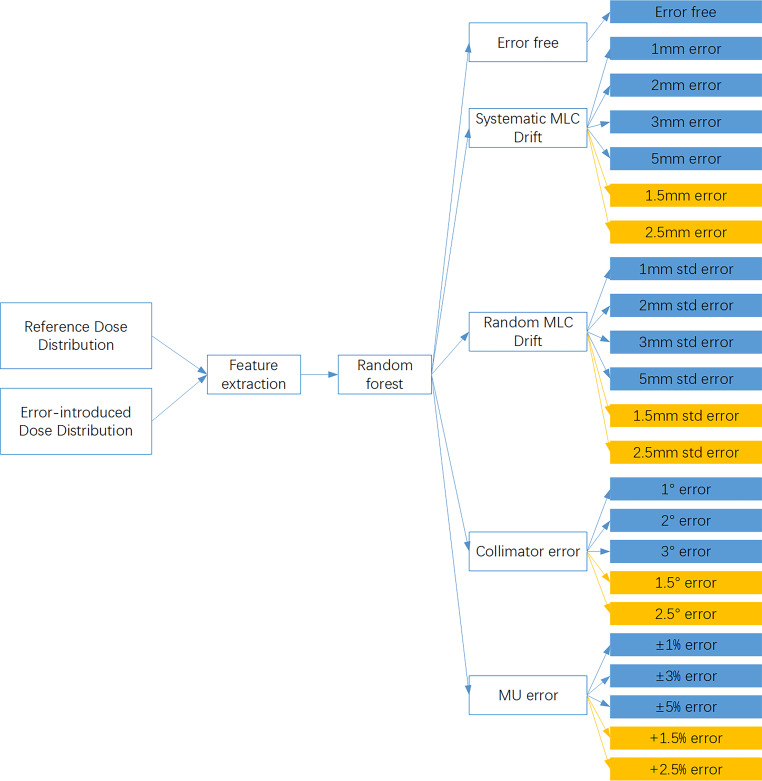
Architecture of this study (training and validation sets are *blue*, testing sets are *yellow*). *MU* monitor units, *MLC* multileaf collimator

The architecture of this study is shown in Fig. 4. The features extracted from RDD and EDD were employed to train the classification model by the random forest algorithm. Meanwhile, the prediction model can be applied to the classification and error magnitude prediction with feature vectors.

The random forest algorithm used in the model was implemented by the scikit-learn library (V0.24.1) of Python (V3.7), based on its default parameter configuration. The datasets for model development were randomly divided into the training set and the validation set at a ratio of 3:1. The random division was repeated 100 times and the average results were calculated. MAE and RMSE calculated as below were utilized to evaluate the performance of this prediction model:

14 $$MAE=\frac{{\sum }_{i=1}^{n}\left| y_{p}\_ \left.y_{a}\right| \right.}{n}$$

15 $$RMSE=\sqrt{\frac{{\sum }_{i=1}^{n}\left(y_{p}-y_{a}\right)^{2}}{n}}$$

where *y*_*p*_ and *y*_*a*_ represent the predicted error magnitude and the actual error magnitude, respectively, *i* and *n* represent the ith field and the total number of fields, respectively.

## Results

### Feature importance

**Fig. 5:**
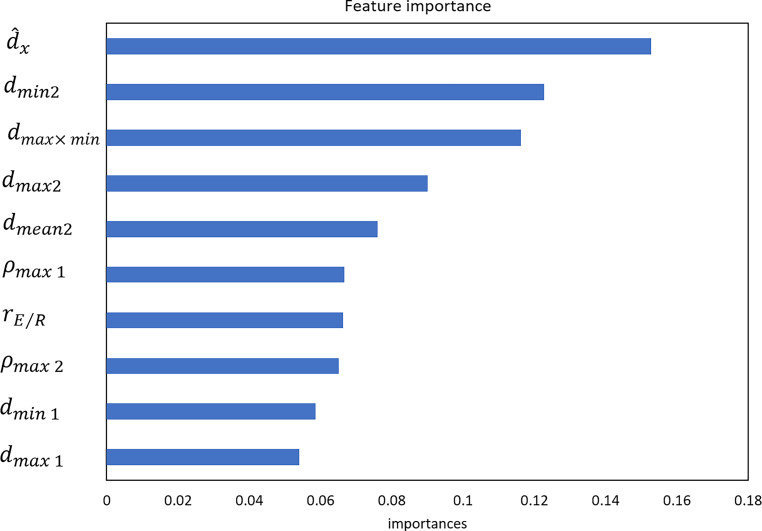
The top 10 features based on their importance ($\hat{d}_{x}$the relative displacement in the x direction; $d_{\mathit{\min }2}$ the ratio of the absolute minimum residual error to the maximal RDD value; $d_{\mathit{\max }\times \mathit{\min }}$ the product of the maximum and minimum residuals; $d_{\mathit{\max }2}$ the ratio of the absolute maximum residual error to the maximal RDD value; $\rho _{\mathit{\max }1}$ the maximum NCC value corresponding to $\hat{\theta }$; *r*_*E*∕*R*_ the ratio between proportion of the maximum gradient direction to all gradient directions in $G\mathrm{'}$ corresponding to EDD and RDD; $\rho _{\mathit{\max }2}$ the maximum NCC value between *T*_*E*_ and *T*_*R*_; $d_{\mathit{\min }1}$ the minimum residual; $d_{\mathit{\max }1}$ the maximum residual)

In this study, a total of 14 features were included in the random forest classification model. This established model can be considered well tuned and effective in distinguishing the error-free condition from other types of errors. In the classification model, the top-ranked features for MLC was the relative displacement in the x direction of EDD and RDD. In terms of the COLL error, the most important feature was the change in the main gradient direction of EDD and RDD. In terms of the MU error, the most important feature was the product of the maximum and minimum residuals. The top 10 features based on their importance as obtained by random forest regression are presented in Fig. 5. The feature importance values were normalized with a range from 0 to 1.0. The top 5 features included the relative displacement in the x direction, the ratio of the absolute value of the minimum residual to the maximum RDD, the product of the maximum and minimum residual, the ratio of the absolute value of the maximum residual to the maximum RDD, and the ratio of the absolute value of the mean residual to the maximum RDD. The relative displacement in the x direction had the highest weight.

**Fig. 6:**
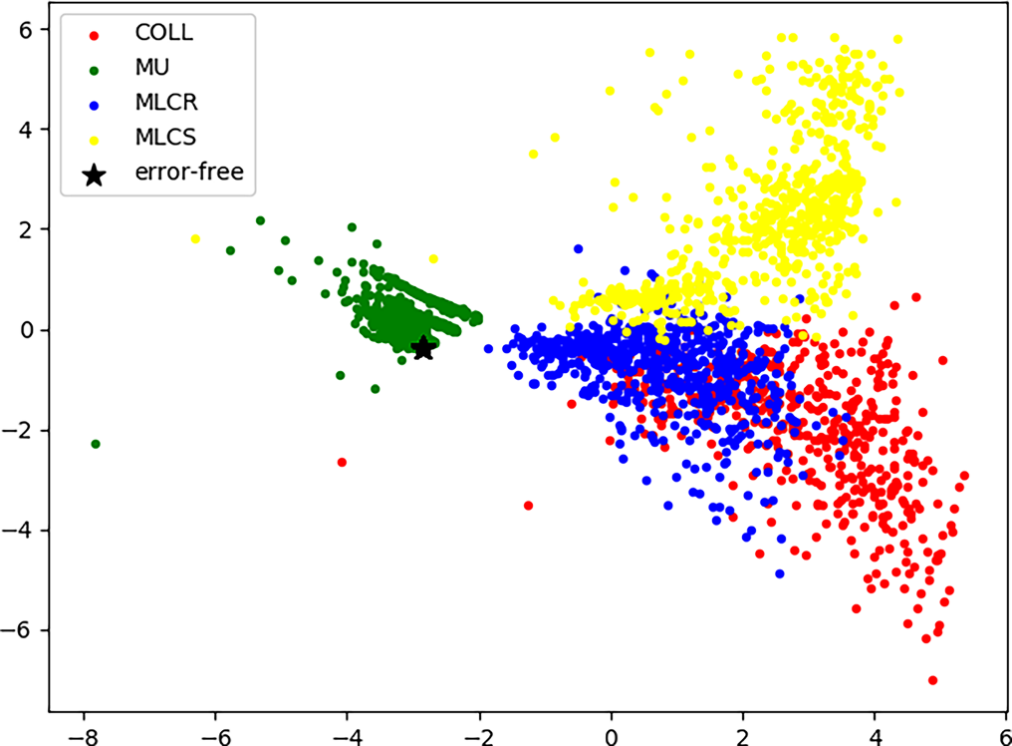
Results of the two-dimensional linear discriminant analysis. *COLL* collimator misalignment, *MU* monitor units variation, *MLCS* systematic multi-leaf collimator misalignment, *MLCR* random MLC misalignment

To obtain some in-depth insights into the relationship between these data, linear discriminant analysis (LDA) was applied for visualization. As shown in Fig. 6, the product of the maximum and minimum residuals and the relative displacement in the x direction were projected down to a two-dimensional scatterplot. The COLL error, MU error, MLCS error, and MLCR error were properly separated into different groups, except for slight overlapping in collimator error and MLCS error. This indicated that the imaged-based features of the four error types were highly distinguishable.

### Accuracy of five-class classification results

**Fig. 7:**
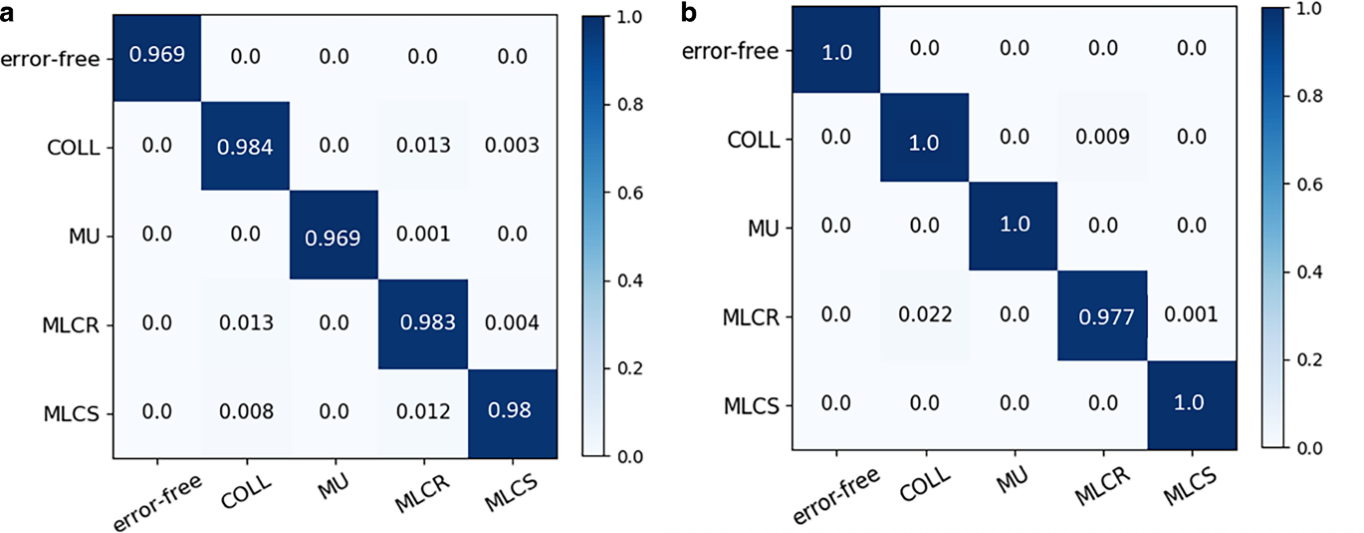
The normalized confusion matrixes of five-class (error-free, collimator error, MU error, MLCR, and MLCS) classification (**a** validation set, **b** test set). *COLL* collimator misalignment, *MU* monitor units variation, *MLCS* systematic multi-leaf collimator misalignment, *MLCR* random MLC misalignment

The classification model based on a random forest showed a favorable performance in error detection and classification, with an overall accuracy reaching 99.85% in the validation set and 99.3% in the testing set. The confusion matrix of five-class experiments for the validation set and testing set are shown in Fig. 7, and the diagonal represents the accuracy of different errors. As for the validation set, this model had the highest accuracy for the error-free condition, with an accuracy reaching 100%. The classification accuracy for the COLL error, MU error, MLCR error, and MLCS error was 98.4%, 99.9%, 98.3%, and 98.0% respectively. In the test set, the classification accuracy for the error-free, COLL error, MU error, MLCR error, and MLCS error was 100.0%, and 97.7% for the MLCR error.

### Classification results of error magnitude

**Fig. 8:**
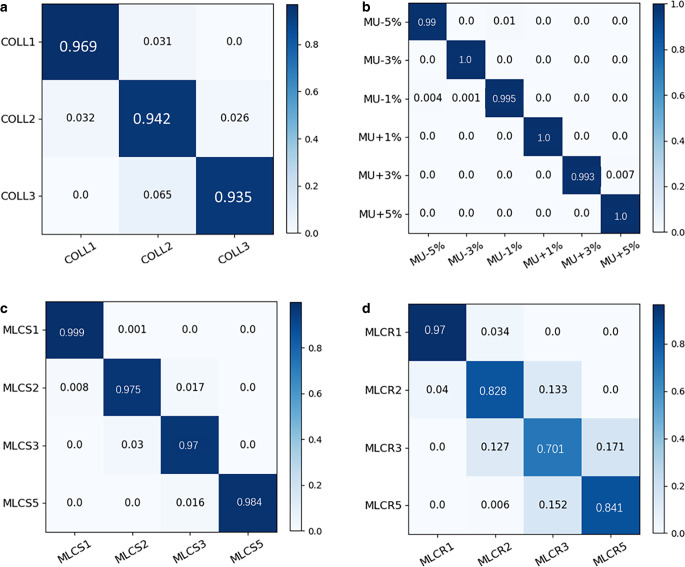
The normalized confusion matrixes of different error magnitude under different error types. **a** COLL errors of 1°, 2°, and 3°; **b** MU errors of ±1%, ±3%, and ±5%; **c** MLCS errors of 1 mm, 2 mm, 3 mm, and 5 mm; **d** MLCR errors of 1 mm, 2 mm, 3 mm, and 5 mm. *COLL* collimator misalignment, *MU* monitor units variation, *MLCS* systematic multi-leaf collimator misalignment, *MLCR* random MLC misalignment

The error magnitude for different errors was classified, and the results were plotted into the confusion matrixes, as shown in Fig. 8. In terms of the COLL error, the accuracy of classification ranged from 93.5% to 96.9%. In terms of the MU error, the classification accuracy was higher than 99%. In terms of the MLCS error, the classification accuracy ranged from 97.0% to 99.9%. In terms of the MLCR error, the accuracy of classification ranged from 70.1% to 96.6%. Among these classifications with different error magnitude levels, MU had the best performance, while MLCR had the worst performance.

### Regression analysis of error magnitude

In the regression analysis of different error magnitudes, the MAE and RMSE of the validation set and testing set were adopted to evaluate the regression error (Table 3). The MAE between predicted error magnitude and actual error ranged from 0.03 to 0.33, with the RMSE varying from 0.17 to 0.56 for the validation set. The MAE and RMSE ranged from 0.38 to 0.50 and 0.44 to 0.59 for the test set, respectively.

**Table 3:** The MAE and RMSE of error magnitude under different error types

	Validation set		Testing set	
	MAE	RMSE	MAE	RMSE
COLL	0.2	0.52	0.43	0.59
MU	0.03	0.19	0.5	0.5
MLCS	0.06	0.17	0.42	0.44
MLCR	0.33	0.56	0.38	0.45

*MAE* mean absolute error, *RMSE* root mean squared error, *COLL* collimator misalignment, *MU* monitor units variation, *MLCS* systematic multi-leaf collimator misalignment, *MLCR* random MLC misalignment

### Gamma analysis for the testing set

Table 4 lists the results of the gamma analysis in the testing set, in which the gamma value decreased with stricter gamma criteria and higher level of error magnitude. However, the gamma analysis could not distinguish different types of error and indicate the error magnitude compared to the machine learning model.

**Table 4:** Gamma result of the testing set (%)

Error type	Error magnitude	3%/3 mm	3%/2 mm	2%/3 mm	2%/2 mm
COLL	1.5°	100.00 ± 0.02	98.14 ± 1.40	100.00 ± 0.03	98.86 ± 1.86
	2.5°	98.06 ± 2.23	91.18 ± 5.33	97.69 ± 2.60	89.63 ± 5.95
MU	1.50%	100 ± 0.00	100 ± 0.00	100 ± 0.00	100 ± 0.00
	2.50%	100 ± 0.00	100 ± 0.00	94.71 ± 6.48	92.16 ± 8.49
MLCR	1.5 mm	99.99 ± 0.04	99.90 ± 0.13	99.98 ± 0.06	99.85 ± 0.16
	2.5 mm	99.70 ± 0.19	98.45 ± 0.59	99.57 ± 0.27	97.97 ± 0.71
MLCS	1.5 mm	100 ± 0.00	100.00 ± 0.00	100.00 ± 0.00	99.99 ± 0.02
	2.5 mm	99.98 ± 0.03	88.29 ± 2.90	99.94 ± 0.06	85.91 ± 3.39

*COLL* collimator misalignment, *MU* monitor units variation, *MLCS* systematic multi-leaf collimator misalignment, *MLCR* random MLC misalignment

## Discussion

In this study, an error classification and error magnitude prediction model was established, including the collimator error, MU error, MLCS error, and MLCR error. The random forest algorithm and error-related features from the RDD and EDD were adopted during model establishment. The overall accuracy of the classification model was 99.85% for the validation set and 99.3% for the testing set. Based on the error-related features, the accuracy of the error detection and classification model in this study was higher than that in previous studies. In the error magnitude prediction for the testing set, the MAE and RMSE of error magnitude for the testing set were 0.38–0.50 and 0.44–0.59, respectively. The error classification and error magnitude prediction model established in this study is expected to become a powerful instrument for IMRT QA.

Although gamma analysis has been extensively applied to patient-specific QA, there are some disadvantages to it. Due to its poor correlation with delivery errors, gamma analysis is considered to be insensitive to some errors [10–12]. Although stricter gamma criteria could achieve better performance in error detection, it is impossible for them to be applicable under all circumstances [24]. In some studies, even 2%/2 mm gamma criteria could not improve the performance of error detection [20]. In addition, it is impossible to determine the specific reason for the failure field of gamma analysis [25]. Although 3D gamma analysis and/or delivery DVHs based on anatomical structure provide more evaluation methods by many 3D verification systems [26], the retrospective error review cannot be performed on plan delivery. The model in this study provides a novel method for error classification and error magnitude prediction in delivery, and it is expected to eliminate the limitations of the well-known conventional IMRT QA method.

During delivery, there may be deviations of actual parameters of collimator, MU, MLC, and gantry from the plan. In this study, the collimator error, MU error, MLCS error, and MLCR error were simulated by modifying corresponding parameters in the original plan. The position of EPID is opposite to the head of the accelerator, and it rotates with the gantry during the treatment of patients, so that EPID cannot measure the effect of gantry error on the planar dose distributions. In this study, the gantry error was not considered. The dose distribution of the error-introduced plan was calculated in this study, different from the QA measurement of dose distribution in other studies. The effectiveness of EDD generated by the calculation method plays an important role in this study. It can be maintained that for a plan without any errors, the actual dose distribution should be similar to RDD. Once the plan with one type of error is delivered, there may be mixed errors, which may affect the features of the specific type of error. Therefore, the same algorithm as RDD was adopted to calculate EDD, rather than measuring the dose distribution of the error-introduced plans, with the aim of learning the specific features of each error. In the selection of error magnitude, it was proposed in the AAPM TG142 report that the tolerance of MU, collimator, and MLC is 1%, 1°, and 1 mm, respectively [27]. In order to explore the accuracy difference among different error magnitude levels, the error in this study included 1°, 2°, or 3° of collimator rotation error. MU variations can reach up to 5%. MLC positioning errors included 1, 2, 3, and 5 mm. In addition, due to the similar processing method, research on the inverse error of collimator and MLC was not included in this study.

In this study, several feature vectors were selected to describe the errors more appropriately from the perspective of image processing. In terms of the collimator angle error, the rotational features were constructed to estimate relative rotational parameters between RDD and EDD. In terms of the MLC error, the translational features were constructed to estimate the relative translational parameters between RDD and EDD. In terms of the MU error, the pixel value change of the EDD was similar to that of the RDD in the effective area. The residual image was obtained by subtracting EDD from RDD, and the gray-level information of the residual image was counted as the relevant feature for evaluating the MU error. Through the analysis of feature importance in the random forest model, it could be found that the most important feature for the collimator angle error was the change of the main gradient direction. As for the MU error, the most important feature was the product of the maximum and minimum residuals. As for MLCR and MLCS errors, the most important feature was the relative displacement in the x direction. The important features of different errors were closely related to the mechanism of error types.

The overall accuracy of the validation and testing set in this study was 99.3%. Wootton et al. performed radiomics analysis on gamma distributions for two-class classification [16]. The AUC of MLC random error was 0.761, and the AUC of MLC systematic error was 0.717. Based on gamma images, Nyflota et al. adopted the DL technique to train the MLC error classification model [17]. The overall accuracy of two-class classification was 77.3%, and the accuracy of three-class classification was 64.3%. Yuto et al. combined dose difference with a convolutional neural network (CNN) to detect the three types of errors in the VMAT plan, with an overall accuracy reaching 94.4% [19]. Ma et al. adopted the SSIM to extract features to perform classification for three types of errors in IMRT, with the highest accuracy reaching 86% [2]. An error detection and classification model with a dual neural network was developed by Potter based on dose-difference histogram (DDH) and distance-to-agreement (sDTA) maps [20]. The accuracy of ANN for two-class classification was 98.3%, and the accuracy of CNN for four-class classification was 95.6%. Different DL architectures and validation methods were adopted in some studies. Although it is difficult to evaluate the relative benefits of one approach versus another, all models could achieve a good performance, which presents a promising prospect. In this study, many new features were extracted from EDD and RDD for radiotherapy QA. The results of this study may further improve the clinical application of the error detection and classification model.

In the classification analysis of error magnitude, as shown in Fig. 8, MLCR has a relatively low accuracy among different magnitude levels. It can be explained by the fact that the ability to capture features is relatively poor due to the uncertainty of leaf errors for the MLCR error. Although MLCR represents a more realistic error condition that merits investigation, the dosimetric effect of random displacement is small [28, 29]. The dose changes introduced by random leaf errors tend to be small, localized clusters [18]. In this study, MLCR was selected as an error type to assess the performance of the proposed method in error detection and classification.

It is important to note that the method proposed in this study is used for dose verification before treatment. As inter- and intrafractional organ motion management is an important task in radiation therapy, Moustakis et al. investigated the feasibility of EPID for online verification of lung SBRT treatment. The method has the potential for in vivo EPID dosimetry in the near future [30]. The prediction is represented in the modeling of this study by including the classification of error types and the prediction of error magnitude, while the “prediction” during treatment means dose distribution prediction based on an algorithm. In this study, the focus is placed on a limited subset of errors, namely only those involving COLL, MU, and MLC. Other types of machine or modeling errors might be more impactful or prevalent in clinical practice, and the method proposed in this study should be expanded to other error types. Besides, the delivery errors involved in this study are not real cases in clinical practice but simulated ones. It is difficult to collect adequate PD images with multiple errors in clinical practice. Since multiple types of errors may occur simultaneously in cases, in which the radiomics features of one type of error are masked by one or more other types of error, it is necessary to construct an error-detection system that can detect mixed errors. This study is limited to thoracic plans of IMRT; future studies will explore the universality of the model to other techniques/sites/plans.

## Conclusion

In this study, image-based features were used in machine learning to achieve discrimination between delivery errors and predict error magnitude for patient-specific IMRT QA. These features can be employed to accurately detect and discriminate the collimator error, MU error, MLCS error, and MLCR error. Besides, they can also be applied to error magnitude prediction. Therefore, it can be expected that they will be applied to patient-specific IMRT QA in the future.

## Supplementary Information

Equations about features to MU error
