# Supplementary material for: Image-based features in machine learning to identify delivery errors and predict error magnitude for patient-specific IMRT quality assurance
Source: Strahlenther Onkol. 2023 Mar 29;199(5):498–510. doi: 10.1007/s00066-023-02076-8 (PMC10133379; doi:10.1007/s00066-023-02076-8)
Supplement: Supplementary file 1 — Equations about features to MU error [file 66_2023_2076_MOESM1_ESM.docx]

The residual image can be calculated as follows,

$I_{D} =I_{E} -I_{R}$ (S-1)

The maximum residual can be calculated as follows,

$d_{\max1}=\max(I_{D} )$ (S-2)

The minimum residual can be calculated as follows,

$d_{\min1}=\min(I_{D} )$ (S-3)

The product of the maximum and minimum residuals can be calculated as follows,

$d_{\max\times\min}=d_{\max1}\times d_{\min1}$ (S-4)

The mean value of residual can be calculated as follows,

$d_{mean1}=\text{mean}(I_{D} )$ (S-5)

The ratio of the absolute maximum residual error to the maximal RDD value can be calculated as follows,

$d_{\max2}=\text{abs}(d_{\max1})/\max(I_{R} )$ (S-6)

The ratio of the absolute minimum residual error to the maximal RDD value can be calculated as follows,

$d_{\min2}=\text{abs}(d_{\min1})/\max(I_{R} )$ (S-7)

The ratio of the absolute mean residual error to the maximal RDD value can be calculated as follows,

$d_{mean2}=\text{abs}(d_{mean1})/\max(I_{R} )$ (S-8)

The ratio of the mean residual error to the mean RDD can be calculated as follows,

$d_{ratio}=\text{mean}(I_{D} )/\text{mean}(I_{R} )$ (S-9)
